# Ensemble Fusion Models Using Various Strategies and Machine Learning for EEG Classification

**DOI:** 10.3390/bioengineering11100986

**Published:** 2024-09-29

**Authors:** Sunil Kumar Prabhakar, Jae Jun Lee, Dong-Ok Won

**Affiliations:** 1 Department of Artificial Intelligence Convergence, Hallym University, Chuncheon 24252, Republic of Korea; sunilprabhakar22@gmail.com; 2 Department of Anesthesiology and Pain Medicine, Hallym University College of Medicine, Chuncheon 24252, Republic of Korea; iloveu59@hallym.or.kr

**Keywords:** EEG, I-ICA, GA, SVM, HHT, KNN

## Abstract

Electroencephalography (EEG) helps to assess the electrical activities of the brain so that the neuronal activities of the brain are captured effectively. EEG is used to analyze many neurological disorders, as it serves as a low-cost equipment. To diagnose and treat every neurological disorder, lengthy EEG signals are needed, and different machine learning and deep learning techniques have been developed so that the EEG signals could be classified automatically. In this work, five ensemble models are proposed for EEG signal classification, and the main neurological disorder analyzed in this paper is epilepsy. The first proposed ensemble technique utilizes an equidistant assessment and ranking determination mode with the proposed Enhance the Sum of Connection and Distance (ESCD)-based feature selection technique for the classification of EEG signals; the second proposed ensemble technique utilizes the concept of Infinite Independent Component Analysis (I-ICA) and multiple classifiers with majority voting concept; the third proposed ensemble technique utilizes the concept of Genetic Algorithm (GA)-based feature selection technique and bagging Support Vector Machine (SVM)-based classification model. The fourth proposed ensemble technique utilizes the concept of Hilbert Huang Transform (HHT) and multiple classifiers with GA-based multiparameter optimization, and the fifth proposed ensemble technique utilizes the concept of Factor analysis with Ensemble layer K nearest neighbor (KNN) classifier. The best results are obtained when the Ensemble hybrid model using the equidistant assessment and ranking determination method with the proposed ESCD-based feature selection technique and Support Vector Machine (SVM) classifier is utilized, achieving a classification accuracy of 89.98%.

## 1. Introduction

One of the most common neurological disorders is epilepsy, and if a timely diagnosis is made for this disorder, then many patients could live their lives without recurrent seizure attacks [1]. For the diagnosis of epilepsy, signal spectrum analysis is quite useful so that different elements like peaks, amplitude, and frequency variation can be known, but at the same time, it could lead to a misdiagnosis if the interpretation of the physician is not correct [2]. In the past three decades, there have been various studies analyzed for the classification of EEG so that various neurological disorders like epilepsy, schizophrenia, dementia, sleep disorders, etc., could be analyzed [3]. The concept of Brain–Computer Interface (BCI), compiled with the advent of machine learning and deep learning, paved the way to obtain a high classification accuracy as it can directly interpret the signal from the individual and can automatically classify the disorder [4]. Previous studies have also highlighted the significance of nonlinear techniques in EEG signals. Hurst exponent, Rhythmicity analysis, Higuchi’s fractal dimension, and Lyapunov exponent are some of the nonlinear techniques that seem to be highly beneficial for the analysis of EEG signals [5]. Certain metrics are utilized so that the origin of the signals is analyzed and the objective of the classification is made clear. To project a good classification, many characteristics are considered, and they can relate to the time domain or frequency domain transformations of the signal. The feature extraction techniques are implemented in ictal, preictal, or interictal periods, depending on the onset of the seizure. Based on the extracted characteristics, the classifiers aim to label and detect epilepsy [6].

Some of the common databases used for epilepsy classification from EEG signals in the past are the Bonn dataset, Bern–Barcelona dataset, own collected dataset, CHB-MIT EEG dataset, The Neurology and Sleep Center of New Delhi dataset, Temple University Hospital (TUH) dataset, etc. [7]. Some prominent works on epilepsy classification from EEG signals in the past few years are discussed as follows. As far as the Bonn dataset is concerned, a rational, discrete, short-time Fourier transform was used by Samiee et al., where a high classification accuracy of 98.10% was obtained [8]. Similarly, a complex-valued neural network with dual-tree complex wavelet transform was used by Peker et al. for Bonn data with a high classification accuracy of 100% [9]. For Bonn dataset, a local binary pattern reported 100% classification accuracy [10]; multiscale radial basis functions with modified Particle Swarm Optimization (PSO) reported 100% classification accuracy [11]; Long Short-Term Memory (LSTM) with SVM reported 99.17% [12]; weighted KNN classifier based on Bray Curtis distance reported 99.67% [13]; phase space representation reported 100% classification accuracy [14], and aforementioned adaptive decomposition methods reported 98.60% classification accuracy [15]. For the Bonn dataset, an affinity propagation congregation-based mutual information with transfer learning reported a classification accuracy of 98.348% [16]; a K-SVD model with Advanced Orthogonal Matching Pursuit (OMP) and Squeeze–Excitation Networks, LSTM, and softmax classifier produced a classification accuracy of 99.56% [17]; Sparse Autoencoder with swarm-based deep learning method and Particle Swarm Optimization (PSO) produced a classification accuracy of 98.55% [18]; sparse representation with swarm intelligence-based Hidden Markov Model (HMM) produced a classification accuracy of 98.94% [19]; and cuckoo search clusters with Linear SVM produced a classification accuracy of 99.48% [20]. For collected epileptic EEG data, a discrete wavelet transform with PSO and Radial Basis Function (RBF) neural network produced a classification accuracy of 99% [21], and for another set of collected epileptic EEG data, the probabilistic neural network with learning vector quantization neural network and Elman neural network produced a classification accuracy of 99.37% [22]. For the CHB-MIT EEG dataset, the concepts of common spatial patterns with CNN were utilized, and a classification accuracy of 90% was obtained [23]. As far as the Bern–Barcelona dataset is concerned, the concept of clustering variational mode decomposition was used, reporting a classification accuracy of 96% [24]; an autoregressive moving average model with SVM was used, reporting a classification accuracy of 99.94% [25], and a locality-sensitive discriminant analysis with SVM was used reporting a classification accuracy of 99% [26]. As far as the TUH database is concerned, four channels were used by techniques such as random forest, KNN, and Principal Component Analysis (PCA) by Lopez et al., and they reported a classification accuracy of 68.30% [27]. Deep CNNs were used for four channels of the TUH database, and a classification accuracy of 79.34% was obtained [28]; nonlinear features with SVM were implemented for four channels of the TUH database, and a classification accuracy of 79.34% was obtained [29]. When 21 channels were utilized in the TUH database, handcrafted features produced a classification accuracy of 85.9% [30]; deep learning produced a classification accuracy of 89.13% [31]; AlexNet with SVM produced a classification accuracy of 87.32% [32], and boosting tree concept produced a classification accuracy of 87.68% [33]. When 24 channels were utilized in the TUH database, deep learning produced a classification accuracy of 85.4% [34], and an improved RNN termed Chrononet produced a classification accuracy of 86.57% [35]. A chaotic local binary pattern with iterative minimum redundancy maximum relevancy was implemented with the TUH database, and a high classification accuracy of 98.19% was obtained for the PZ channel; 97.46% was obtained for the O2 channel; 97.82% was obtained for the C4 channel; 95.65% was obtained for the F4 channel; 96.74% was obtained for the O1 channel, and 97.46% was obtained for the T5 channel [36]. 

The main contributions of this work are as follows. As a basic pre-processing step, Independent Component Analysis (ICA) is used, and then the proposed models are implemented.

(a) The first proposed ensemble technique utilizes an equidistant assessment and ranking determination mode for the classification of EEG signals;
(b) The second proposed ensemble technique utilizes the concept of Infinite Independent Component Analysis (I-ICA) and multiple classifiers with a majority voting concept;
(c) The third proposed ensemble technique utilizes the Genetic Algorithm (GA)-based feature selection technique and bagging SVM-based classification model;
(d) The fourth proposed ensemble technique utilizes the concept of Hilbert Huang Transform (HHT) and multiple classifiers with GA-based multiparameter optimization;
(e) The fifth proposed ensemble technique utilizes the concept of Factor analysis with an Ensemble layer K nearest neighbor (KNN) classifier.

The organization of this paper is as follows. In Section 2, the proposed ensemble techniques are explained, and the results of the discussion are explained in Section 3, and this paper ends with the conclusion in Section 4.

## 2. Proposed Ensemble Techniques

The five proposed ensemble techniques are explained in detail as follows.

### 2.1. Proposed Technique 1: Ensemble Hybrid Model Using Equidistant Assessment and Ranking Determination Method

The design of the ensemble methodology is described as follows:

The procedure comprises three important steps:

(a) Equidistant assessment of the basic model parameters;
(b) K-means clustering with ranking assessment and determination is utilized for ensemble pruning;
(c) The final prediction result is voted on with the help of the divide-and-conquer strategy.

#### 2.1.1. Equidistant Assessment of the Model Parameters

**Figure 1:**
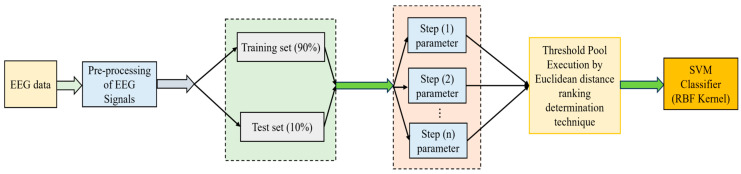
Ensemble hybrid model using equidistant assessment and ranking determination method with SVM Classifier.

The independent training of every basic model takes place, and the equidistant assessment of the parameters is performed. The idea of the ensemble model is to develop multiple basic models and then integrate them [37]. In this work, certain models $m=\left\{m_{1},\ldots ,m_{i},\ldots ,m_{22}\right\}$ are used so that a set of basic models is generated, which includes classifiers such as SVM, LR, KNN, RF, NB, DT, and various other learning models. In this work, only binary class issues are dealt with in detail. Assume Ω={0,1} is a collection of binary class labels; assume $\vec{z}={ℜ}^{n}$ is a vector that has n features that would be embedded and labeled Ω. The training set is considered as $H=\left\{\left({\vec{z}}_{1},l_{1}\right),\left({\vec{z}}_{2},l_{2}\right),\ldots ,\left({\vec{z}}_{n},l_{n}\right)\right\}$, where $l_{i}\in \Omega$. Once the training set D is randomly sorted, 90% of D is selected as the training set, and 10% of D is selected as a test set. In two different stages, the basic models are constructed. The sets are trained horizontally when the basic structure of every model is built. The parameters of these models are optimized when the basic model is trained. When the parameter assessment is finished, every optimal parameter is saved. The ensemble hybrid model using the equidistant assessment and ranking determination method with the SVM classifier is shown in Figure 1.

The steps are described as follows: A random sort on the set is performed, and then it is split into a train set and a test set. The base structure of the model $m_{i}$ is built with the help of a training set. The parameters of the models $m_{i}$ are optimized in equidistant assessment. For instance, for a given $m_{i}$, the model chosen is KNN; then, a parameter k is possessed by that model, which is nothing but the total number of weights to be utilized. The optimal parameters are defined as “k, 2, 16, 4”. It implies that the K value ranges from 2 to 16, using four steps for these optimal parameters. This implies that these four parameter-equidistant assessment tasks are created. To train the model, a corresponding value is employed by each task so that the performance is evaluated using the validation set. After that, the four tasks are aligned to the thread pool as it has a kind of multithreading unit. To obtain the desired output, the number of threads can be set automatically or manually. The submitted tasks would be running in parallel with the thread pool so that the best parameter is saved. In this work, four threads are used in $m_{i}$, so that the parameter of each model is optimized. The equidistant assessment optimization is expressed in Algorithm 1.
**Algorithm 1:** Equidistant assessment optimizationInput: model $m_{i}$ and parameter φ (“k, 2, 16, 4”)Disintegrate φ and obtain each step $k_{i}$ value.for *i* = 1 to step doAdd every step task into the thread pool.end forTrain the modelSave $m_{i}$ in equidistant mode.

#### 2.1.2. Evaluation Assessment for Ranking Determination

To reach an important decision in ensemble research, a technique must be chosen that is quite diverse among the hybrid of multiple basic models. To assess the diversity among the models, many statistics are involved in it. A parameter of inter-rater agreement k is considered, and the diversity value k is assessed as follows:

(1) $$k=1-\frac{1}{2\vec{q}\left(1-\bar{q}\right)}Dis_{av}$$

where $\vec{q}$ and $Dis_{av}$ is expressed as follows:

(2) $$\vec{q}=\frac{1}{nt}\sum_{j=1}^{n}\sum_{i=1}^{t}m_{i}\left(z_{i},l_{j}\right)$$

(3) $$Dis_{av}=\frac{t}{t\left(t-1\right)}\sum_{i=1}^{t}\sum_{k=1,i\neq k}^{t}Dis_{i,k}$$

$Dis_{av}$ represents the average distance.

#### 2.1.3. Design of Ranking Determination Method

An approach called ranking determination is introduced in the proposed works. Compared to other selection methods, the chosen technique is quite flexible and robust. A stopping condition is used in hierarchical selection so that exhaustive search is avoided [38]. The values 0 and 1 are chosen to indicate whether the model is selected for the membership of the ensemble model. The vector $\vec{z}=\left\{1,0,\ldots ,1\right\}$ is the identified hybrid model of the ensemble idea. The candidate basic models are pruned by the k-means algorithms before the ranking determination technique [39]. The models are partitioned into subsets so that the k-means algorithm can be employed in subsets. The data are randomly divided into the train set and the test set. The input of the k-means algorithms is nothing but the prediction outcomes of the validation of every model. If the validation data comprise v instances, and the result of the prediction class by the model $m_{i}$ is $w^{i}=\left\{w_{1},w_{2},\ldots ,w_{v}\right\}$, these output values are used by k-means to compute the Euclidean distance by every model. The Euclidean distance of models $m_{i}$ and $m_{j}$ for the $z^{th}$ instance is defined as $d_{ijz}$. There are only two values, 0 or 1, for $w_{i}$, and if the original prediction of the $z^{th}$ instance by $m_{i}$ is 0, which is similar to the prediction result by $m_{j}$; then $d_{ijz}=0$ or else, it becomes $d_{ijz}=1$. The Euclidean distances of $m_{i}$ and $m_{j}$ are computed as $\sum_{k=1}^{k=v}d_{ijk}$. The value of k is assigned as 20, so the basic model is partitioned into 20 clusters. The best performing model is chosen from every subset, and the procedure is continued. The ensemble framework acts as a primary base for the ranking determination. The selected combination of the basic model is chosen by the vector $m_{i}$ of the solution. Whether to accept a new solution is decided by the algorithm, and the probability is selected dynamically and updated. Ultimately, the best hybrid model is selected and evaluated. For every basic model, the selection probability is chosen as 0.4. A solution is generated initially, which is composed of 0 s and 1 s. Using ranking determination, the solution of the ultimate combination is chosen. A new solution is generated in the second layer, and using a simulated annealing algorithm at every hierarchy, the process is analyzed. In the new solution, the selection probability of every model is increased. The probability values of the basic model are updated dynamically once the algorithm is updated.

#### 2.1.4. Ensemble Hybrid Technique

The prediction results of the basic models are combined into a hybrid so that a good generalization ability is achieved. The division of a combination vote is categorized into weighted and unweighted votes. For the unweighted vote, the most popular hybrid technique is a majority vote. It traces $h_{opt}$, as it is widely chosen by various models. Assuming that the class of $z_{i}$ is $h_{opt}$, the class set of data is $h_{opt}=\left\{0,1\right\}$, as only a binary class is considered. The output class label $h_{opt}$ of the ensemble model is defined as follows:

(4) $$h_{opt}=\{\begin{array}{cc} 1 & if\;\sum_{i=1}^{v}m_{i}\left(z_{j}\right)\geq \frac{1}{2}v\\ 0 & otherwise \end{array}$$

where the number of basic models is v.

With the help of vote weights, the output class label of the ensemble model $h_{opt}$ is defined as follows:

(5) $$h_{opt}=\{\begin{array}{cc} 1 & if\;\sum_{i=1}^{v}w_{i}m_{i}\left(z_{j}\right)\geq \frac{1}{2}v,w_{i}\geq 0\sum_{i=1}^{v}w_{i}=1\\ 0, & otherwise \end{array}$$

A large voting task is initially divided into two small tasks. The time complexity is computed for the model. If the prediction number instance is huge, then the ensemble voting would be time-consuming if the single thread approach is utilized. A hybrid vote strategy was employed so that the ensemble voting time could be saved. Then, for every majority vote task, the threshold is set. A large ensemble voting task is recursively divided into two smaller tasks if the prediction instance is higher than the specified threshold value. Therefore, the larger task is divided into many smaller tasks, and then it is submitted to a thread pool. Ultimately, for all small tasks, the prediction result is conquered, thereby mitigating the time complexity. If there are a higher number of hybrid models, more time can be saved. 

#### 2.1.5. Feature Selection

The performance must be improved by eliminating redundant features, and that is achieved through feature selection. To manage high dimensional data, feature selection is highly utilized. Here, a technique called “Enhance the Sum of Connection and Distance” (ESCD) is proposed. To assess the independence of every feature, a distance function is utilized. In a subset, to measure the relevance between the features, the Pearson Correlation Coefficient (*PCC*) is utilized so that the redundancy among the features is computed [40]. To introduce ESCD, some notations are provided. The input data are expressed as $D=\left\{\left({\vec{z}}_{1},l_{1}\right),\left({\vec{z}}_{2},l_{2}\right),\ldots ,\left({\vec{z}}_{n},l_{n}\right)\right\}$, where the target class is expressed as $l_{i}$, and there are total Q features $F=\left\{f_{1},where\begin{array}{cc} i=1,\ldots ,Q & \end{array}\right\}$. Finding a subspace of q features ${ℜ}^{q}$ is the main intention and is chosen from the M-dimensional original space ${ℜ}^{q}$. The linear correlation of the two variables is reflected by *PCC*. To assess the relevance between a target class and a feature, *PCC* is chosen. For a target class label vector $\vec{l}$ and feature vector $\vec{F}$, the *PCC* is computed as follows:

(6) $$PCC\left(\vec{F},\vec{l}\right)=\frac{\frac{1}{N-1}\sum_{h=1}^{N}\left(f_{h}-\bar{f}\right)\left(l_{h}-\bar{l}\right)}{\sqrt{\frac{1}{N-1}\sum_{h=1}^{N}{\left(f_{h}-\bar{f}\right)}^{2}}\sqrt{\frac{1}{N-1}\sum_{h=1}^{N}{\left(l_{h}-\bar{l}\right)}^{2}}}$$

(7) $$\bar{f}=\frac{1}{N}\sum_{k=1}^{N}f_{h}$$

(8) $$\bar{l}=\frac{1}{N}\sum_{k=1}^{N}l_{h}$$

where the hth elements of $\vec{F}$ and $\vec{l}$ are represented by $f_{h}$ and $l_{h}$.

The connection value of the feature is expressed as follows:

(9) $$C_{i}=\left|PCC\left({\vec{F}}_{i},\vec{l}\right)\right|\left(1\leq i\leq Q\right)$$

To obtain a high classification performance, there should be minimal redundancy. Among the various features, the similarity level is assessed by using distance metrics. As Euclidean distance is quite easy to understand and interpret, it is used widely. The computation of Euclidean distance between two features is calculated as follows:

(10) $$ED\left({\vec{F}}_{i},{\vec{F}}_{j}\right)=\sqrt{\sum_{k=1}^{N}{\left(f_{ih}-f_{jh}\right)}^{2}}\left(1\leq i,j\leq Q,i\neq j\right)$$

The Euclidean Distance (ED) value of the feature i is expressed as follows:

(11) $$D_{i}=\frac{1}{Q-1}\sum ED\left({\vec{F}}_{i},{\vec{F}}_{h}\right),\left(1\leq h\leq Q,h\neq i\right)$$

The condition that hybridizes $D_{i}$ with $C_{i}$ is called ESCD, and the selection condition is expressed as follows: $\max\left(C_{i}+D_{i}\right)$. Here, the connection is the same as the distance. A good improvement in the prediction accuracy is obtained by the ensemble method compared to a single method. The proposed ESCD feature selection technique is compared with other conventional feature selection techniques like GA, Particle Swarm Optimization (PSO), Ant Colony Optimization (ACO), and Glowworm Swarm Optimization (GSO). Experimental results show that the proposed ESCD feature selection technique surpasses the performance of the conventional feature selection techniques when dealing with the ensemble hybrid model and classified with a Support Vector Machine Classifier (SVM), a classifier utilizing the Radial Basis Function (RBF) kernel.

### 2.2. Proposed Technique 2: Ensemble Hybrid Model Using Infinite I-ICA and Multiple Classifiers with Majority Voting Concept

#### 2.2.1. Feature Extraction and Selection Using Infinite ICA

For a feature space $z\in {ℜ}^{N}$, a mapping is found by the feature selection technique $w=f(z):{ℜ}^{N}\to {ℜ}^{M}\left(M<N\right)$, so that the vital information of z is preserved by the transformed feature vector $w\in {ℜ}^{M}$. Various feature selection techniques are present in the literature so that informative feature sets can be extracted. To obtain the independent and uncorrelated features, these statistical feature extractors are used. With the help of ICA, the observed data z are transformed with a linear transformation H into independent components w as z=Hw+e, where the Gaussian noise is indicated as e [41]. With reference to the dimension reduction technique, these schemes have been utilized for feature selection. There is no inference to the dimensionality of the novel feature vector, and the determination of the new features must be made in advance. So, to solve this problem and to obtain the total number of independent features from input, an extension of ICA called infinite ICA is used. To mask a hidden source w, the assessment of a binary vector b is made, so that the activity of the hidden source is shown by its elements and is represented as

(12) Z=H[W⊙B]+E

where Z,W,B,E indicate the concatenation of ${\left\{z_{i}\right\}}_{i=1}^{N},{\left\{w_{i}\right\}}_{i=1}^{N},{\left\{b_{i}\right\}}_{i=1}^{N}$, and ${\left\{e_{i}\right\}}_{i=1}^{N}$, respectively. The element-wise multiplication is denoted by ⊙. An infinite number of hidden sources is obtained as B has many rows. For Q hidden sources and D data points, the distribution of matrix B is expressed as

(13) $$p\left(B|\pi_{1},\ldots ,\pi_{Q}\right)=\pi_{q=1}^{Q}\pi_{i=1}^{D}P\left(b_{q_{i}}|\pi_{q}\right)=\pi_{q=1}^{Q}\pi_{q}^{m_{q}}{\left(1-\pi_{q}\right)}^{D-m_{q}}$$

where $b_{q_{i}}$ specify activity of qth source for a particular sample using probability of $\pi_{q}$, and $m_{q}=\sum_{i=1}^{D}b_{q_{i}}$ indicates the total number of active sources. A Gaussian noise has been analyzed, so that E could be defined with a particular variance $\sigma_{e}^{2}$. Ultimately, for informing W hidden sources from Z observed data, H mixing matrix is used. Gibbs sampling technique is implemented for sampling elements with $b_{q_{i}}=1$. Using the Bayes rule, the conditional distribution of one parameter is always sampled. By choosing more informative features, classification becomes less complex, and real-time computation becomes easier.

#### 2.2.2. Random Subspace Ensemble Learning Classification

Only a small training inset has been utilized by most existing techniques, which does not match the high dimensionality of the problem. As a result, the feature-to-instance ratio becomes quite large. A low classification performance is obtained when training the classifier utilizing the small training dataset with high dimensionality. A novel classification method called the random subspace ensemble technique with SVM as the base classifier is proposed so that the computational load is reduced. The feature space is $z\in {ℜ}^{N}$, and the mapping $w=f(z):{ℜ}^{N}\to {ℜ}^{M}\left(M<N\right)$ is found out by feature selection technique I-ICA. The random subsets in the feature space are utilized by random subspace, and the total subspaces are denoted by $r_{1},\ldots ,r_{s}$. On every subspace, the implementation of SVM classifiers is performed so that the input data can be classified. With the help of majority voting, the output with a large number of votes is selected. To prevent overfitting, which is caused by small size data and high dimensionality, ensemble learning schemes are considered instead of single classifiers [42]. The overall learning performance is improved, as these schemes provide a collective decision by weak hybrid classifiers. A random sample of features is used in the proposed random subspace ensemble technique, and the selection of features is made randomly and assigned to the classifier.

#### 2.2.3. Ensemble Learning in a Random Manner

The randomly selected feature subspace learns all the decision rules in every SVM classifier. The D dimensional output of I-ICA is divided initially into S subspaces randomly and indicated by $r_{1},\ldots ,r_{s}$. On every subspace, SVM is implemented for the classification of input data. Mapping of the input space is performed onto a high dimensional feature space by a nonlinear function Φ(r). The decision function is expressed as

(14) f=sgn(v·Φ(r)−b)

sgn indicates the sign of a real number, and b indicates a bias. A hyperplane is identified so that the features of the two classes are separated, and it is represented as $\frac{2}{\left\|w\right\|}$. Thus, a quadratic function is maximized with respect to their linear constraints. The solving of quadratic programming is performed as follows:

(15) $$v\left(\alpha \right)=0.5{\left\|v\right\|}^{2}-\alpha \left[O(v\cdot \Phi (r)-b)-1\right]$$

where a collection of Lagrange multiplier is expressed as α. To minimize the classification error, Gaussian RBF is used. This Kernel RBF is represented as follows:

(16) $$k\left(r_{i},r_{j}\right)=\Phi^{T}(r_{i})\cdot \Phi \left(r_{j}\right)=\exp\left\{{\left\|r_{i}-r_{j}\right\|}_{2}^{2}/2\sigma^{2}\right\}$$

where the two feature vectors are represented by $r_{i}$ and $r_{j}$. The squared Euclidean distance is represented by ${\left\|r_{i}-r_{j}\right\|}_{2}^{2}$. The output with the highest number of votes is found out by majority voting and represented as the final output of the system. To enhance generality, cross validation is used, as the correlation strength does not correlate directly with the new observation. For a specific statistical model, to find the predictive accuracy, Cross Validation is also used. 

#### 2.2.4. Random Ensemble Learning by Hybrid Classifiers

**Figure 2:**
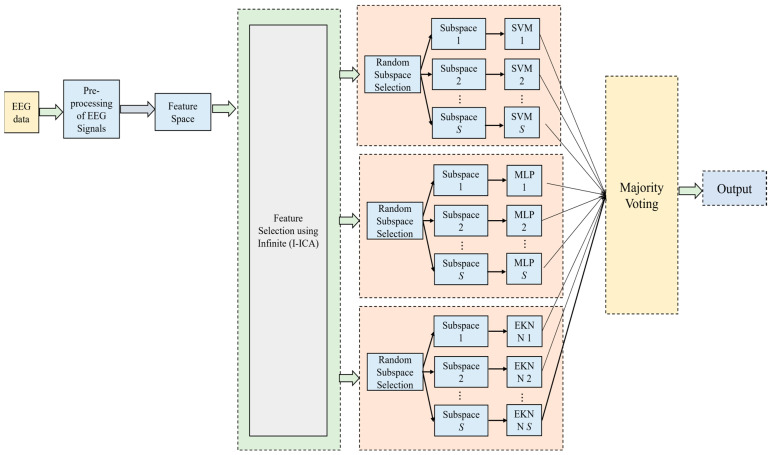
Ensemble hybrid model using Infinite I-ICA and multiple classifiers with majority voting concept.

Hybrid classification models are considered in our work. Multilayer Perceptron (MLP) neural network, Extended KNN (EKNN), and SVM are utilized together in this hybrid model. A two-layer feed-forward neural network with one hidden layer is used in the MLP classifier, and a sigmoid is used as an activation function. For training the network, various optimization techniques are used, and finally, the backpropagation technique is utilized. To code the output, a mathematical hard limit function is utilized. To trace and identify the derivative of the loss, a backpropagation algorithm is used, so that the weight optimization is performed successfully. The gradient component for every weight is computed by using the error function. In the EKNN technique, the neighboring test samples adjacent to the classes are analyzed comprehensively so that the classification accuracy is improved [43]. Here, in an iterative manner, the unknown sample is assigned to every class so that the class membership can be easily predicted. The ensemble hybrid model using Infinite I-ICA and multiple classifiers with a majority voting concept is shown in Figure 2.

### 2.3. Proposed Technique 3: Ensemble Hybrid Model with GA-Based Feature Selection and Bagging SVM-Based Classification Model

**Figure 3:**
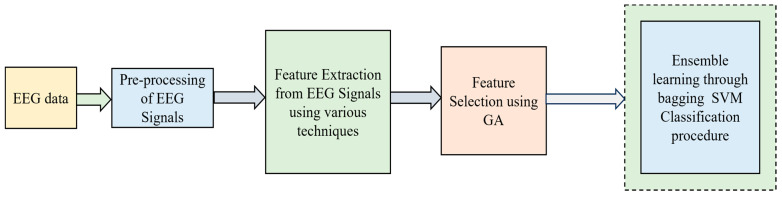
Ensemble hybrid model with GA-based feature selection and bagging SVM-based classification model.

The extracted features included wavelet transforms, Fast Fourier transforms (FFTs), fractal dimension, mobility, peak amplitude, complexity, zero crossing, variance, frequency band power, skewness, mean, kurtosis, correlation coefficient, average spectral power, entropy, energy, line length, detrend fluctuation analysis, absolute value sum, Hurst exponent, entropy, amplitude, etc. The Ensemble hybrid model with GA-based feature selection and bagging SVM-based classification model is shown in Figure 3.

#### 2.3.1. Genetic Algorithm

One of the famous parallel stochastic search optimization techniques is GA [44]. Initial populations are generated randomly, and the population is updated continuously through genetic operators to derive a better solution. The feature parameters are coded initially, and then the population of the initial size is assessed. In the entire population, every individual finds a possible solution. Depending on the fitness value function, the fitness value of every individual is computed. For the entire population, the probability of mutation and crossover is set. Unless a certain criterion is met by the population performance by the genetic strategies such as selection rate, crossover rate, and mutation rate, the process is continued. The procedure must fulfill a certain number of iterations to meet the performance of the population. The basic steps are as follows:

(1) The modeling of every feature is performed as a gene, and almost all the features are like chromosomes, as they share a similar length to the features. A varied subset of features is indicated by every chromosome;
(2) The highest evolution algebra A is set. The initial population is created that includes all the N individuals;
(3) Every individual is projected as $P_{q}^{1},P_{q}^{2},\ldots ,P_{q}^{N},q=0$;
(4) In every chromosome, the number “1” is generated randomly, and then the random assignment of these chromosomes is performed so that a varied number of features can be clearly represented;
(5) The evaluation value of fitness is tested, and the main intention of feature selection is utilized with fewer features, so that a good classification rate can be achieved;
(6) The feature subset input and the classification accuracy are used to evaluate the fitness function for every individual and are represented as follows:
  (17) F(Pqi)={ReAcFno(min),i=1,…,N
  where the recognition accuracy is represented by ReAc, and $F_{no(\min)}$ indicates the number of features. The features traced in the feature subset are used when the classifiers are trained;
(7) To select the operators, roulette is used, implying that based on fitness ratio, the chromosomes are selected. The probability of chromosomes is represented as follows:
  (18) $$\mathrm{Pr}=F_{i}/\sum_{i=1}^{N}F_{i},i=1,2,\ldots ,N$$
  where $F_{i}$ indicates the reciprocal of fitness value, and the population size is indicated by N;
(8) The single-point cross technique is utilized, and two individuals are chosen with similar probabilities from $P_{q}^{1},P_{q}^{2},\ldots ,P_{q}^{N}$. Unless a new group is formed, this process is repeated;
(9) Based on a particular mutative probability, the value of every individual is randomly changed, and a new generation of groups is created, such as $P_{q+1}^{1},P_{q+1}^{2},\ldots ,P_{q+1}^{N}$;
(10) Check whether the termination condition is satisfied or not. If the condition is satisfied, the entire operation stops as the best solution with a high fitness value is obtained as output. Otherwise, step 2 is repeated once again.

Based on statistical learning theory, a famous machine learning technique proposed is SVM. The main principle of it is as follows: Given a training set $T=\left\{\left(p_{i},q_{i}\right)|i=1,2,\ldots ,n\right\}$ for a two-class problem, where $p_{i}\in P$ is n dimension feature vectors in the real number field, $q_{i}\in \left\{-1,+1\right\}$, whose values are −1 or 1. To indicate the hyperplane, a linear equation $w^{T}p+b=0$ is used if the training set is linearly separable. The normal vector is indicated by $w=\left(w_{1},w_{2},\ldots ,w_{n}\right)$, which helps to assess the hyperplane direction. The distance between the origin and hyperplane is indicated by constant b. The distance from every point p in the sample space to the hyperplane is expressed as $\gamma =\frac{\left|w^{T}p+b\right|}{\left\|w\right\|}$. In the optimal classification hyperplane, the kernel function $k\left(p_{i},p_{j}\right)$ is utilized to replace the dot product so that the classification performance of SVM is optimized. The classification threshold of the sample is expressed as $b^{*}=q_{i}-\sum_{i=1}^{l}q_{i}\alpha_{i}^{*}K\left(p_{i},p_{j}\right)$, where $\alpha_{i}$ indicates the Lagrange multiplier. The ultimate discriminate function is expressed as

(19) $$f(p)=\mathrm{sgn}\left(w\cdot p+b\right)=\mathrm{sgn}\left\{\sum_{i=1}^{n}q_{i}\alpha_{i}^{*}K(p_{i},p_{j})+b^{*}\right\}$$

#### 2.3.2. Ensemble Learning through Bagging Procedure

To improve the generalization performance of the classifiers and to enhance the accuracy of the learner, an ensemble learning technique dependent on bagging-SVM is proposed [45]. To obtain multiple training subsets, ensemble learning utilizes such datasets generally. The subset of data is trained by each base classifier, and then, all these base classifiers are hybrid so that a novel ensemble classifier is created. In the incremental data, by means of utilizing a self-sampling technique, the training set is extracted by using an SVM-based bagging algorithm. The change in the novel information is reflected by the development of an ensemble classifier so that the novel sample sets can be made quite different from each other. Then, each subsample set is learned by using multiple SVM classifiers, and then, the majority vote technique is used for the learning so that ensemble incremental learning is implemented. The main implementation of the Bagging SVM algorithm is performed as follows: Assuming an aggregate as B, the Rth round of self-help sampling for the aggregate B is carried out. From aggregate B(r=1,2,…,R), the Rth subsets $B_{t}$ comprising the Qth sample. For a base classifier, by utilizing the SVM algorithm, the new training sample $B_{r}$ is learned. Then, a weak classifier $\varphi \left(p,B_{k}\right)$ is produced by every subset $B_{r}$, and the error rates of weak classifiers are computed $\varphi \left(p,B_{k}\right)$:

(20) $$\varepsilon_{t}=\Sigma_{\left(p_{i},q_{i}\right)\in B_{r}}\left[\varphi \left(p,B_{k}\right)\neq q_{i}\right]/\left|B_{t}\right|$$

The training set $B_{r+1}$ is extracted independently again based on some distribution. Then, the integration of the Rth weak classifier into a strong classifier Φ(p,B) is performed, and the ultimate decision function is obtained. The strong classifier Φ(p,B) outputs the voting results when entering the test sample for the rth weak classifiers $\varphi \left(p,B_{k}\right)$. This implies that in the test sample categories, there would be the majority categories in the voting process. For every base classifier, a comprehensive predictive result is present. A higher performance is achieved by the Bagging algorithm than the traditional single classifier, as far as the prediction classification analysis is concerned.

### 2.4. Proposed Technique 4: Ensemble Hybrid Model with HHT and Multiple Classifiers with GA-Based Multiparameter Optimization

In this method, HHT is implemented initially [46]. At the outset, the FFT of the input signal is computed. The coefficients of the FFT with respect to the negative frequencies are made negligible, and then the inverse FFT is computed. The definition of HT is expressed as follows:

(21) $$y(t)=\frac{1}{\pi }PV\int_{-\infty }^{\infty }\frac{x\left(t^{\prime }\right)}{t-t^{\prime }}dt$$

where the Cauchy principal value is represented by PV. Once the pre-processing of the EEG signal is made, Empirical Mode Decomposition (EMD) is performed, so that the data can be decomposed into the intrinsic mode functions using the sifting process. The instantaneous frequencies from the Instantaneous Mode Functions (IMFs) are attained; then, the Hilbert spectrum is performed, and finally, the feature vector is formed.

#### 2.4.1. Random Forest Regression Model

One of the famous supervised learning techniques is RF [47]. It is a hybrid model, which encompasses a regression decision subtree. The regression prediction result is nothing but the mean of every decision subtree based on the principle of ensemble learning. The vital steps of this algorithm are as follows: 

The random generation of the s training sets $\theta_{1},\theta_{2},\ldots ,\theta_{s}$ is made using the bootstrap resampling technique. A decision tree is generated by every training set, where the number of random forests of the tree is denoted as s and is indicated as

(22) $$\left\{T\left(p,\theta_{1}\right)\right\},\left\{T\left(p,\theta_{2}\right)\right\},\ldots ,\left\{T\left(p,\theta_{s}\right)\right\}$$

The random extraction of ‘f’ features is performed from F dimensional feature samples in the process of node splitting, and, based on the sample size, ‘f’ is set. To enhance its growth, no pruning is made for every decision tree. The prediction of a single decision tree T(θ) is obtained when there are novel data P=p. By means of averaging the leaf node values l(p,θ) it is obtained. If $P_{i}$ belongs to the leaf node l(p,θ) and is not zero, then the weight vector $w_{i}\left(p,\theta \right)$ is expressed as

(23) $$w_{i}\left(p,\theta \right)=\frac{l\left\{P_{i}\in R_{i}\left(p,\theta \right)\right\}}{\neq \left\{j=P_{j}\left(p,\theta \right)\right\}}$$

For a single decision tree, the predicted value is the weighted average of the predicted values $Q_{i}\left(i=1,2,\ldots ,n\right)$ for an independent variable P=p. For a single decision tree, the predicted value is expressed as

(24) $$\bar{\mu }=\sum_{i=1}^{N}w_{i}\left(p,\theta \right)Q_{i}$$

The decision tree weights are averaged as P=P(i∈(1,2,…,n)); the weight $w_{i}(p)$ is attained for every observation i∈(1,2,…,S) as

(25) $$w_{i}(p)=\frac{1}{s}\sum_{i=1}^{s}w_{i}\left(p,\theta \right)q$$

For all q, the recording of the prediction of the random forest is implemented as $\bar{\mu }$ and expressed as

(26) $$\bar{\mu }=\sum_{i=1}^{N}w_{i}(p)Q_{i}$$

#### 2.4.2. LightGBM Model

Dependent on tree learning, a famous gradient learning framework is the LightGBM model [48]. The accuracy is much higher, and the training efficiency is faster when compared with XGBoost. This model is adaptable to various optimization problems. The continuous floating-point eigenvalues are discretized into K integers using this framework, and then a histogram-based decision tree algorithm is constructed. To ensure high efficiency, a leaf-wise leaf growth strategy optimization algorithm is proposed with a depth limitation, so that over-fitting is prevented.

#### 2.4.3. XGBoost Model

One of the most popular algorithms for ensemble learning is the boosting algorithm. For every weak classifier, the weights are combined, superimposed, and hybridized, so that a strong classifier is formed, so that the error is reduced, and accuracy is improved. A significant improvement in the boosting method is gradient boosting. The residuals are incessantly reduced, and then the residual of the previous model is also reduced in the direction of the gradient so that a new model is obtained. For the loss function, a second order Taylor expansion is implemented by XGBoost, and thus, an optimal solution is obtained [49]. The main steps are as follows:

The objective function is as follows:

(27) $$F\left(\phi \right)=\Sigma_{i}l\left({\hat{q}}_{c},q_{i}\right)+\Sigma_{h}\in \left(f_{h}\right)$$

(28) $$\mathrm{where}\;\in \left(f_{h}\right)=\gamma T+\frac{1}{2}\alpha {\left\|w\right\|}^{2}$$

A differentiable convex loss function is represented by c that analyzes the difference between the target $q_{i}$ and prediction ${\hat{q}}_{c}$. The complexity of the model is penalized by ∈; the number of tree ensemble is represented by H; the number of learning in the tree is represented by T; γ and α are expressed as proportion. 

The objective function training is calculated as follows:

(29) $$F^{t}=\sum_{i=1}^{n}c\left(q_{i},{\hat{q}}^{\left(t-1\right)}+f_{t}\left(p_{i}\right)+\varepsilon \left(f_{t}\right)\right)$$

The prediction of the ith instance at tth iteration is represented by ${\hat{q}}^{\left(t\right)}$, so that the objective function is minimized. The approximation of the objective function using Taylor two-order expansion is achieved and the optimal solution for the objective function is obtained. 

#### 2.4.4. Ensemble Technique Dependent on GA-Based Multiparameter Optimization

**Figure 4:**
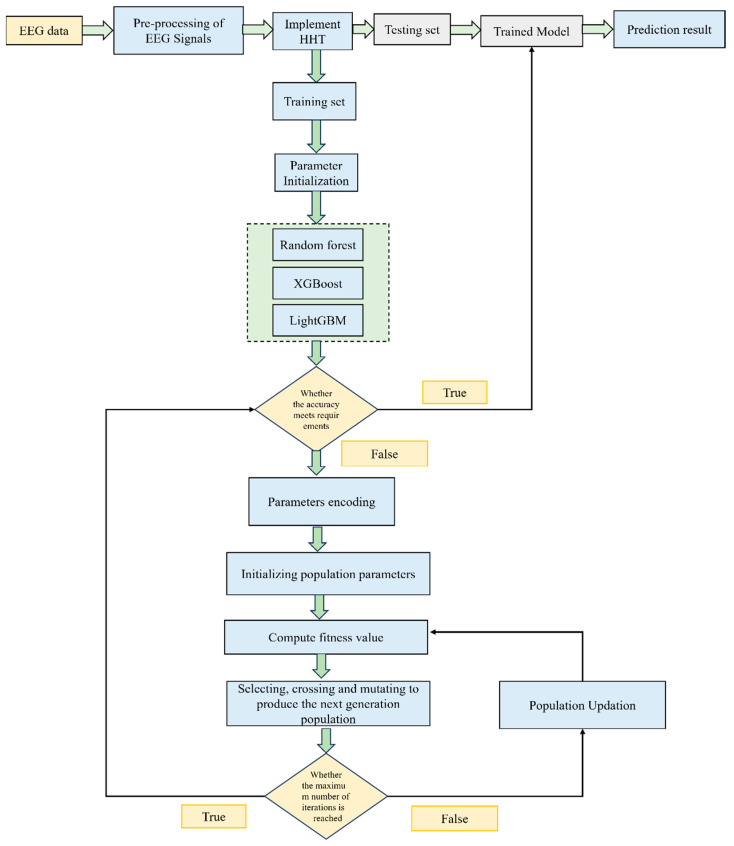
Ensemble hybrid model with HHT, multiple classifiers, and GA-based multiparameter optimization.

As far as the RF model is concerned, the hyperparameters have a huge impact on the prediction results. The parameters are the number of features used by a single decision tree, depth of the decision tree (maximum and minimum), number of leaf nodes (maximum and minimum), number of samples (maximum and minimum), etc. For the XGBoost algorithm, the learning rate, leaf node, sample weight, and depth of the tree are the important hyperparameters. As far as the LightGBM model is concerned, the number of leaf nodes (minimum and maximum), learning rate, tree depth model, and leaf node weight (minimum and maximum) are quite important. To optimize these parameters, the traditional grid search takes too much time, so a genetic algorithm is performed, as it has a strong global search capacity. Once the data preprocessing is finished, the original data are split into a train set and a test set. The parameters of the GA, such as crossover, population size, and mutation probability, are initialized. The optimization parameters are selected. The parameters to be optimized are chosen from these machine learning models, and the determination of the optimal interval is executed by chromosome coding. The fitness function is determined, and the average relative error between the true value and the predicted value is computed. The chromosomes in the population are decoded, and then the fitness value of every generation is computed. Then, the survival of the fittest is performed. The optimal parameters are obtained as an output if the population performance manages the highest number of genetics. The optimization would be over if the error requirement is satisfied. The best prediction result is obtained by inputting the test sample. The ensemble hybrid model with HHT, multiple classifiers, and GA-based multiparameter optimization is shown in Figure 4.

### 2.5. Proposed Technique 5: Ensemble Hybrid Model with Factor Analysis Concept and Ensemble-Layered KNN Classifier

#### 2.5.1. Factor Analysis

**Figure 5:**
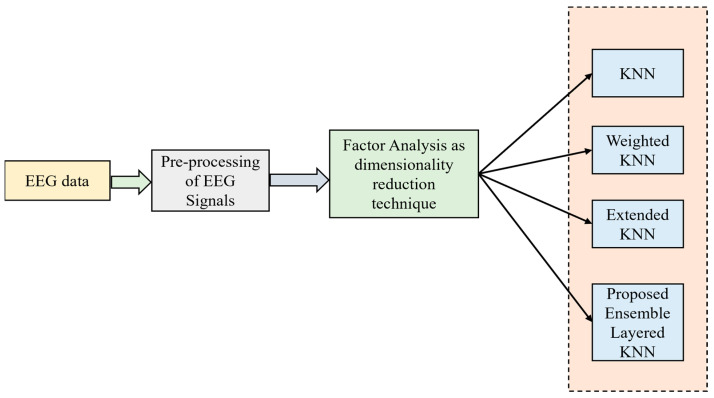
Ensemble hybrid model with Factor analysis concept and ensemble-layered KNN classifier.

A famous statistical method related to PCA is factor analysis [50]. It is a generative model, where the observed data are assumed to be produced from a collection of latent unobserved variables termed as factors with the help of an equation x=WX+n. If noise is absent, then this model could be considered as PCA, though PCA is not generative in nature. All the variances of the respective factors are imbibed into W in this technique, so that the covariance of X serves as the identity matrix. A multivariate novel distribution is followed by the factors, and it is totally uncorrelated to the noise. Under such a condition, the observed variables covariance is written as follows:

(30) $$C_{x}=WW^{t}+C_{n}$$

where the covariance matrix of the noise is indicated as $C_{n}$ and must be estimated from the data. By matrix factorization of $WW^{t}=C_{x}-C_{n}$, the matrix W is solved completely. The ensemble hybrid model with Factor analysis concept and ensemble-layered KNN classifier is shown in Figure 5.

#### 2.5.2. Layered K-Nearest Neighbor Classifier

The proposed LKNN has three vital steps such as outlier rejection, training phase, and testing phase, respectively [51]. In the initial step, using the Interquartile range (IQR), the rejection of the stray items is achieved using an effective technique. IQR is a simple assessment of the concentration of data and can analyze the data spread quickly. IQR and median serve as quite versatile measures like mean and standard deviation. The outliers can be rejected for every target by tracing the class center, initially using the following equation:

(31) $$Center(c_{i})=\left\{\frac{\sum_{g=1}^{ee}u_{g}^{1}}{ee},\frac{\sum_{g=1}^{ee}u_{g}^{2}}{ee},\ldots ,\frac{\sum_{g=1}^{ee}u_{g}^{n}}{ee}\right\}$$

where the employed feature space in the center of $c_{i}$ is denoted as $Center(c_{i})$; the total number of examples that belong to $c_{i}$ is denoted as ee, and the value of the ith dimension of the gth example is denoted as $u_{v}^{i}$. 

The distance from every class member to the center of the class is computed accordingly. Between the center $c_{i}$ and an input $I_{h}$, the Euclidean distance is specified as $Dis\left(I_{h},c_{i}\right)$. In between the points ${\vec{U}}_{x}^{i}$ and ${\vec{U}}_{y}^{i}$, the Euclidean distance in the m dimensional feature space is computed using the following equation:

(32) $$Dis\left({\vec{U}}_{x}^{i},{\vec{U}}_{y}^{i}\right)=\sqrt{\sum_{i=1}^{m}{\left(u_{x}^{i}-u_{y}^{i}\right)}^{2}}$$

where the values of the ith dimensions of ${\vec{U}}_{x}^{i}$ and ${\vec{U}}_{y}^{i}$ are represented as $u_{x}^{i}$ and $u_{y}^{i}$, respectively. The computations are highly influenced by the outliers. IQR is quite efficient against other influences. No distributions are allowed by the data with respect to the IQR. With the help of Equation (32), the distance from the center of the class to every class member is computed, and it is split into four portions such as Q1, Q2, Q3, and Q4, respectively. The smallest quarter of values is covered by Q1, while the highest quarter of values is covered by Q4. The mid-half of the dataset that is present between Q1 and Q4 is defined by IQR. Therefore, IQR = Q3-Q1, where Q1 is the ith quartile. The computation of two values (higher and lower) is carried out, which are expressed as $V_{low}$ and $V_{high}$, respectively.

(33) $$V_{low}=Q_{1}-1.5\times (IQR)$$

(34) $$V_{high}=Q_{1}+1.5\times (IQR)$$

The data points presented below $V_{low}$ are assigned as class expressive items, while the data points presented above $V_{high}$ are assigned as outliers. The training stage is quite an important step in the LKNN classifier as it sets a process termed as Layers Construction Process (LCP). Let us assume q target classes, $Q=\left\{q_{1},q_{2},q_{3},\ldots ,q_{n}\right\}$, and for every target class, $q_{i}\forall q_{i}\in Q$, the computation of the distance from its center $\left(c_{i}\right)$ to the item, which is further from its members, are computed and specified as $F_{far}^{i}$. The specification of the maximum distance is calculated as follows:

(35) $$F_{\max}=\underset{\forall q_{i}\in Q}{\max}\left(F_{far}^{i}\right)$$

The computation of layer width is performed as $\frac{F_{\max}}{L}$, where the arbitrary number of layers is expressed by L. The organization of every class is performed in cascaded layers, depending on the computed layer width. The number of class layers will be higher if the distance from the class center is further. The computation of the weight of every layer is performed using weight(layeri)=Li, where the large number is specified by i, and the number of layers is specified by L. The original classification takes place considering the need to classify a new item $N_{j}$ into one of the respective classes; $Q=\left\{q_{1},q_{2},q_{3},\ldots ,q_{t}\right\}$. At the initial stage, $N_{j}$ is specified as the m-dimensional feature space. The identification of the KNN is achieved by the collection set $C_{KNN}$. In between the novel item and every existing item, the degree of association is expressed as $N_{k}\forall N_{k}\in C_{KNN}$. In between the input item $N_{j}$ and an arbitrary item $N_{k}\in C_{KNN}$, the association rate is expressed as

(36) $$\underset{\forall N_{k}\in C_{KNN}}{AR\left(N_{j},N_{k}\right)}=\frac{1}{Dis\left(N_{j},N_{k}\right)}$$

where $Dis\left(N_{j},N_{k}\right)$ is the distance between the input $N_{j}$ and an arbitrary item $N_{k}\in C_{KNN}$.

For the ‘t’ target class,

(37) $$C_{KNN}=C_{q_{1}}\cup C_{q2}\cup C_{q3}\ldots \cup C_{q_{t}}$$

(38) $$\mathrm{where}\;\;\;C_{q_{i}}=\left\{N_{m}|N_{m}\in C_{KNN},N_{m}\in q_{i}\right\}$$

The novel item $N_{j}$ has an affiliation degree to a target class $q_{i}$ and is expressed as $AD\left(N_{j},q_{i}\right)$; it is written as follows:

(39) $$AD\left(N_{j},q_{i}\right)=\sum_{\forall N_{m}\in C_{q_{i}}}\left[AR\left(N_{j},N_{m}\right)\times weight\left(N_{m}\right)\right]$$

where $AD\left(N_{j},q_{i}\right)$ represents the affiliation degree of the novel item $N_{j}$ to the class $q_{i}$. The association rate between the input item $N_{j}$ and $N_{m}$ is represented by $AR\left(N_{j},N_{m}\right)$. The input item $N_{j}$ belonging to the class $q_{i}$ is used to determine classification decision and is expressed as

(40) $$classification\left(N_{j},q_{i}\right)=\frac{AD\left(N_{j},q_{i}\right)}{\sum_{\forall q_{m\in }Q}AD\left(N_{j},q_{m}\right)}$$

The target class of $N_{j}$ specified as Target $\left(N_{j}\right)$ is expressed as

(41) $$T\arg et\left(N_{j}\right)=\underset{\forall q_{i}\in Q}{\arg\max}\left[Classification\left(N_{j},q_{i}\right)\right]$$

## 3. Results and Discussion

To detect epilepsy seizures, an EEG database called TUH is utilized in this paper [52]. There are two classes (normal and abnormal) in this database, and it has the details of 18,000 patients and comprises nearly 28,000 files. The age range of the patients in this database is around 16 to 90 years. The acquisited EEG signals are stored in. edf format, and the EEG signals were collected using 10/20 recordings. The clinical history of every patient, along with the medication summary, is contained in their database. The details of the subjects used for this study are as follows. As far as the training set is considered, the normal subjects of females were around 691, and the normal subjects of males were around 546. The abnormal subjects of females were around 454, and the abnormal subjects of males were around 439. As far as the testing set is considered, the normal subjects of females were around 84, and the normal subjects of males were around 64. The abnormal subjects of females were around 51, and the abnormal subjects of males were around 54, respectively.

As far as the experimental setup is concerned, a basic computer with 64 GB RAM, 512 GB solid state disk, windows 10 operating system, and a 3.2 GHz microprocessor were used in this work and implemented using MATLAB 2022a. For GA used in the experiment, the hyperparameters are set as follows. The population size is set as 300; the number of generations is set as 250; elitism is set at 10; crossover probability is set as 0.6; mutation probability is set as 0.2, and the number of iterations is set at 500. For RF Classifier used in this experiment, the default number of estimators is 100, and the max_depth is set as 50. The min_samples_split is set as 25; min_samples_leaf is set as 15, and the max_leaf_nodes is set as 10. For XGBoost and LightGBM classifiers used in this experiment, the eta value is set as 0.4; gamma is set as 0.2; max_depth is set as 4; min_child_weight is set as 2; max_delta__step is set as 1.5, and lambda and alpha values are set as 1, respectively. A 10-fold cross-validation technique was used when implementing it with machine learning classifiers. All 24 channels were utilized in the TUH dataset, and for performance evaluation, sensitivity, specificity, and accuracy are analyzed in this work as follows:

(42) $$Sensitivity=\frac{TP}{TP+FN}$$

(43) $$Specificity=\frac{TN}{TN+FP}$$

(44) $$Accuracy=\frac{TP+TN}{TP+TN+FP+FN}$$

where TP denotes True Positive; FP indicates False Positive; TN specifies True Negative, and FN denotes False Negative, respectively. Table 1 shows the results of the ensemble hybrid model using the equidistant assessment and ranking determination method with the proposed ESCD-based feature selection technique. It is evident that a high classification accuracy of 89.98% is obtained with the proposed method, while the lowest classification accuracy of 83.84% is obtained if ACO is used as a feature selection technique and classified with an SVM classifier. Table 2 shows the performance analysis of I-ICA with random ensemble learning by hybrid classifiers, and it is evident that a high classification accuracy of 89.5% is obtained, and a comparatively low classification accuracy of 82.88% is obtained only if I-ICA is used with MLP classifier. Table 3 shows the performance analysis of GA with bagging SVM, and the results show that a high classification accuracy of 88.15% is obtained, and a low classification accuracy of 82.89% is obtained if GA is analyzed with Linear SVM. Table 4 shows the performance of the ensemble hybrid model with HHT and multiple classifiers with GA-based multiparameter optimization; a high classification accuracy of 89.96% is obtained, and a comparatively low classification accuracy of 85.23% is obtained if the analysis is tried with the LightGBM classifier alone. Table 5 shows the performance of the ensemble hybrid model with the Factor analysis concept and ensemble-layered KNN classifier; a high classification accuracy of 88.61% is obtained, and a comparatively low classification accuracy of 81.22% is obtained if the concept of factor analysis is dealt with KNN ensemble hybrid model alone. 

**Figure 6:**
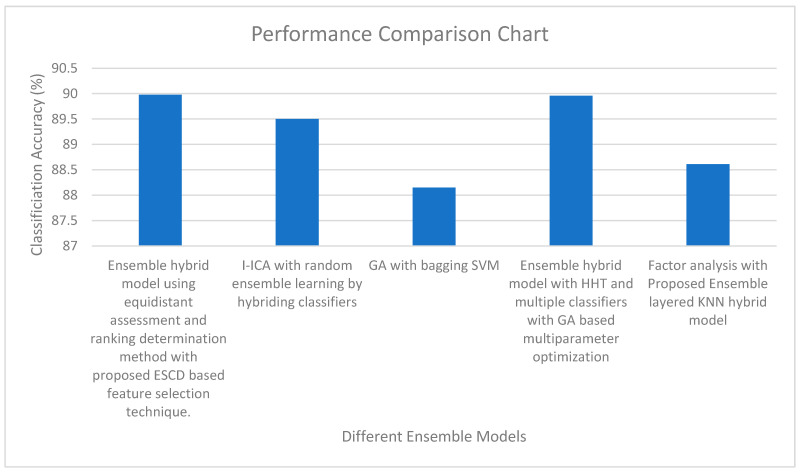
Performance Comparison of different ensemble models.

Figure 6 shows the performance comparison of various ensemble models. It is quite evident from Figure 6 that the highest classification accuracy is obtained by the ensemble hybrid model using the equidistant assessment and ranking determination method with the ESCD-based feature selection technique. The second highest classification accuracy is obtained by the ensemble hybrid model with HHT and multiple classifiers with GA-based multiparameter optimization. The lowest classification accuracy is obtained by the GA with the bagging SVM classifier. Table 6 shows the performance comparison of the current work with the results of the previous works.

On analyzing Table 6, it is evident that the highest classification accuracy of 89.98% is obtained for the ensemble hybrid model using the equidistant assessment and ranking determination method with the proposed ESCD-based feature selection technique. The intrinsic properties of the hybrid algorithms can be attributed to the success of obtaining a high classification accuracy. The second-best classification accuracy of 89.96% is obtained for the ensemble hybrid model with HHT and multiple classifiers with GA-based multiparameter optimization. Almost all the proposed works perform well when compared to the previously obtained results except the results reported in [36], where the researchers have concentrated on individual channels separately. The Ensemble hybrid model using equidistant assessment and ranking determination method with proposed ESCD-based feature selection technique and SVM classifier produces a computational complexity of $O\left(n^{3}\log n\right)$; I-ICA with random ensemble learning by hybrid classifiers produces a computational complexity of $O\left(n^{3}\log n\right)$; GA with bagging SVM produces a computational complexity of $O\left(n^{4}\log2n\right)$; Ensemble hybrid model with HHT and multiple classifiers with GA-based multiparameter optimization produces a classification accuracy of $O\left(n^{5}\log4n\right)$, and Factor analysis with Proposed Ensemble-layered KNN hybrid mode produces a computational complexity of $O\left(n^{3}\log n\right)$.

The interpretability of the decisions of the proposed models can greatly aid the clinical applications as good classification accuracy has been obtained in this work. Future works aim to explore a plethora of other interesting techniques so that the classification accuracy can be increased even more. The practical applicability and utility of the proposed methods can be implemented in test beds so that they can be successfully incorporated into clinical settings. It could even be implemented for remote health care monitoring services in the future with the aid of cloud-based application development.

## 4. Conclusions and Future Works

The activities of the brain can be easily assessed with the help of EEG. To the different types of visual stimuli, the human brains respond in a unique and different manner, and these responses can be analyzed well with the help of EEG. Analyzing EEG signals, machine learning, and deep learning are highly useful. The EEG features can be extracted in the time domain/frequency domain/spatial domain, and then it is fed into the classifiers when dealing with the machine learning techniques, whereas deep learning techniques learn the features on their own. Deep learning also utilizes end-to-end Artificial Neural Networks (ANN), so that the machines can automatically extract and the signal features can be easily filtered. In this work, five ensemble models are proposed for the classification of epilepsy from EEG signals. All five proposed ensemble models produced a classification accuracy of more than 85%, and future works aim to incorporate a variety of other interesting hybrid ensemble models so that the classification accuracy could be increased. The main advantage of using hybrid models is that the intrinsic properties of a variety of algorithms could be utilized efficiently so that the overall versatility of the system could be greatly improved. Also, future works will aim to develop and extend the implementation of cloud-based telemedicine applications. 

## Figures and Tables

**Table 1:** Ensemble hybrid model using equidistant assessment and ranking determination method with proposed ESCD-based feature selection technique.

Techniques Proposed	Sensitivity (%)	Specificity (%)	Accuracy (%)
Ensemble hybrid model using equidistant assessment and ranking determination method with GA-based feature selection method and SVM Classifier.	85.45	86.45	85.95
Ensemble hybrid model using equidistant assessment and ranking determination method with ACO-based feature selection method and SVM Classifier.	84.34	83.34	83.84
Ensemble hybrid model using equidistant assessment and ranking determination method with PSO-based feature selection method and SVM Classifier.	85.34	85.46	85.4
Ensemble hybrid model using equidistant assessment and ranking determination method with GSO-based feature selection method and SVM Classifier.	86.36	87.51	86.93
Ensemble hybrid model using equidistant assessment and ranking determination method with proposed ESCD-based feature selection technique and SVM Classifier.	88.98	90.99	89.98

**Table 2:** Performance analysis of I-ICA with random ensemble learning by hybrid classifiers.

Techniques Proposed	Sensitivity (%)	Specificity (%)	Accuracy (%)
I-ICA with SVM classifier	86.23	85.34	85.78
I-ICA with MLP classifier	82.34	83.43	82.88
I-ICA with EKNN classifier	87.65	88.32	87.98
I-ICA with random ensemble learning by hybrid classifiers	89.99	89.01	89.5

**Table 3:** Performance analysis of GA with bagging SVM.

Techniques Proposed	Sensitivity (%)	Specificity (%)	Accuracy (%)
GA with Linear SVM	83.45	82.34	82.89
GA with Polynomial SVM	85.45	85.01	85.23
GA with Radial Basis Function Kernel SVM	87.77	86.99	87.38
GA with bagging SVM	88.01	88.29	88.15

**Table 4:** Ensemble hybrid model with HHT and multiple classifiers with GA-based multiparameter optimization.

Techniques Proposed	Sensitivity (%)	Specificity (%)	Accuracy (%)
Ensemble hybrid model with HHT and RF classifier with GA-based multiparameter optimization	88.03	87.91	87.97
Ensemble hybrid model with HHT and LightGBM classifier with GA-based multiparameter optimization	86.23	84.23	85.23
Ensemble hybrid model with HHT and XGBoost classifier with GA-based multiparameter optimization	87.23	87.11	87.17
Ensemble hybrid model with HHT and multiple classifiers with GA-based multiparameter optimization	90.01	89.91	89.96

**Table 5:** Ensemble hybrid model with Factor analysis concept and ensemble layered KNN classifier.

Techniques Proposed	Sensitivity (%)	Specificity (%)	Accuracy (%)
Factor analysis with KNN ensemble hybrid model	82.21	80.23	81.22
Factor analysis with Weighted KNN ensemble hybrid model	83.34	83.45	83.39
Factor analysis with Extended KNN ensemble hybrid model	84.45	85.43	84.94
Factor analysis with Proposed ensemble-layered KNN hybrid model	88.21	89.01	88.61

**Table 6:** Performance Comparison of average classification accuracy of the current results with the previous works.

References	Techniques Used	Number of Channels Used	Classification Accuracy (%)
Lopez et al. [27]	Ensemble learning with KNN and RF	4	68.30
Sharma et al. [29]	Nonlinear features with SVM	4	79.34
Yildrim et al. [28]	Deep CNN	4	79.34
Gemein et al. [30]	Handcrafted features	21	85.9
Alhussein et al. [31]	Deep learning	21	89.13
Amin et al. [32]	AlexNet and SVM	21	87.32
Albaqami et al. [33]	Boosting concept	21	87.68
Schirrmeister et al. [34]	Deep learning	24	85.4
Roy et al. [35]	Chrononet	24	86.57
Tuncer et al. [36]	Concept of Chaotic Local binary pattern with iterative minimum redundancy maximum relevancy	PZ Channel	98.19
Proposed works 1	Ensemble hybrid model using equidistant assessment and ranking determination method with proposed ESCD-based feature selection technique and SVM classifier	24	89.98
Proposed works 2	I-ICA with random ensemble learning by hybrid classifiers.	24	89.5
Proposed works 3	GA with bagging SVM	24	88.15
Proposed works 4	Ensemble hybrid model with HHT and multiple classifiers with GA-based multiparameter optimization	24	89.96
Proposed works 5	Factor analysis with Proposed Ensemble-layered KNN hybrid model.	24	88.61

## Data Availability

Publicly available data were used for this work. The dataset can be obtained and referred from “I. Obeid, J. Picone, The Temple University Hospital EEG data corpus, Frontiers in neuroscience, 10 (2016), p. 196”.
